# Supplementary figures and images for: Evaluation of the efficacy of using indocyanine green associated with fluorescence in sentinel lymph node biopsy
Source: PLoS One. 2023 Oct 25;18(10):e0273886. doi: 10.1371/journal.pone.0273886 (PMC10599532; doi:10.1371/journal.pone.0273886)

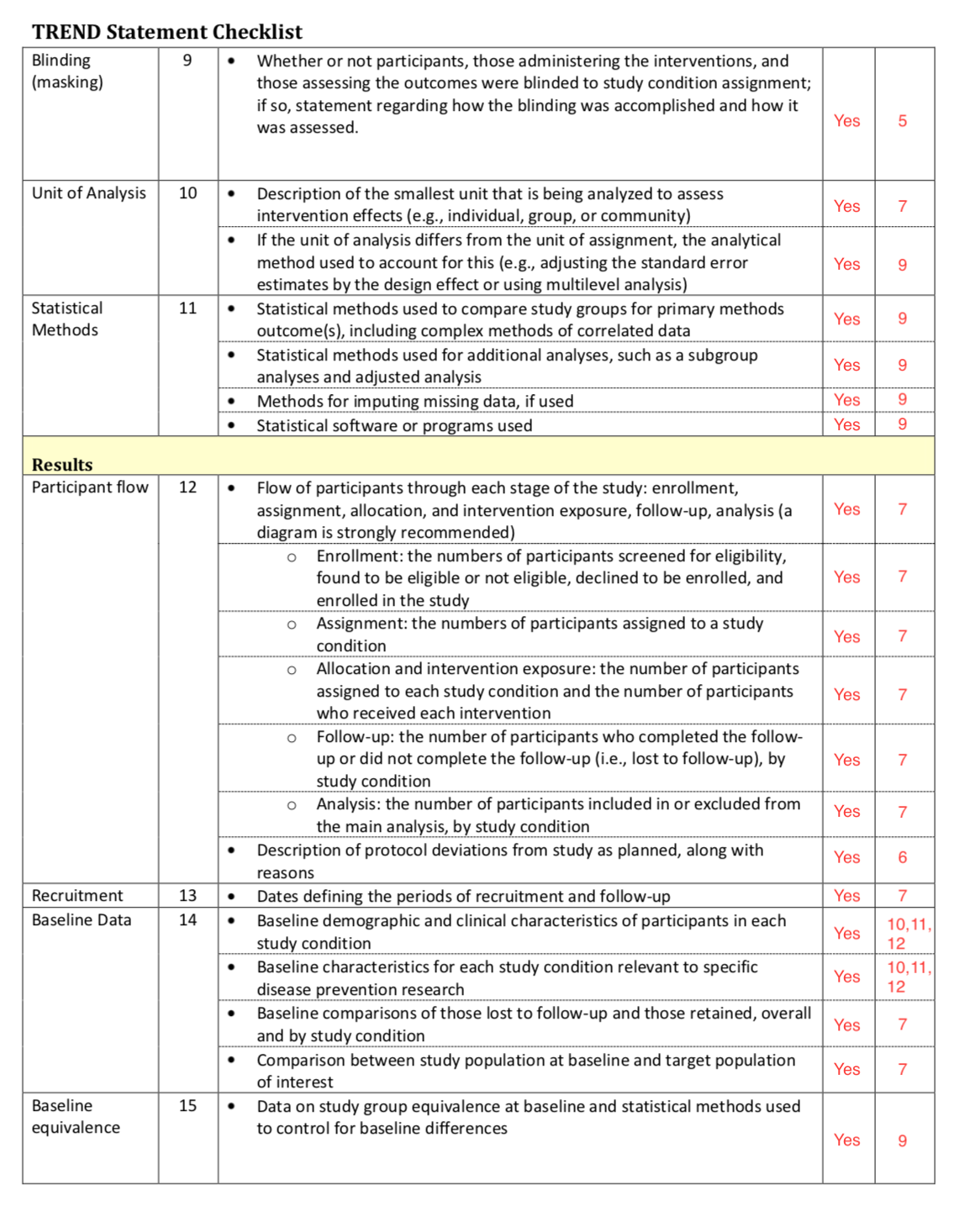


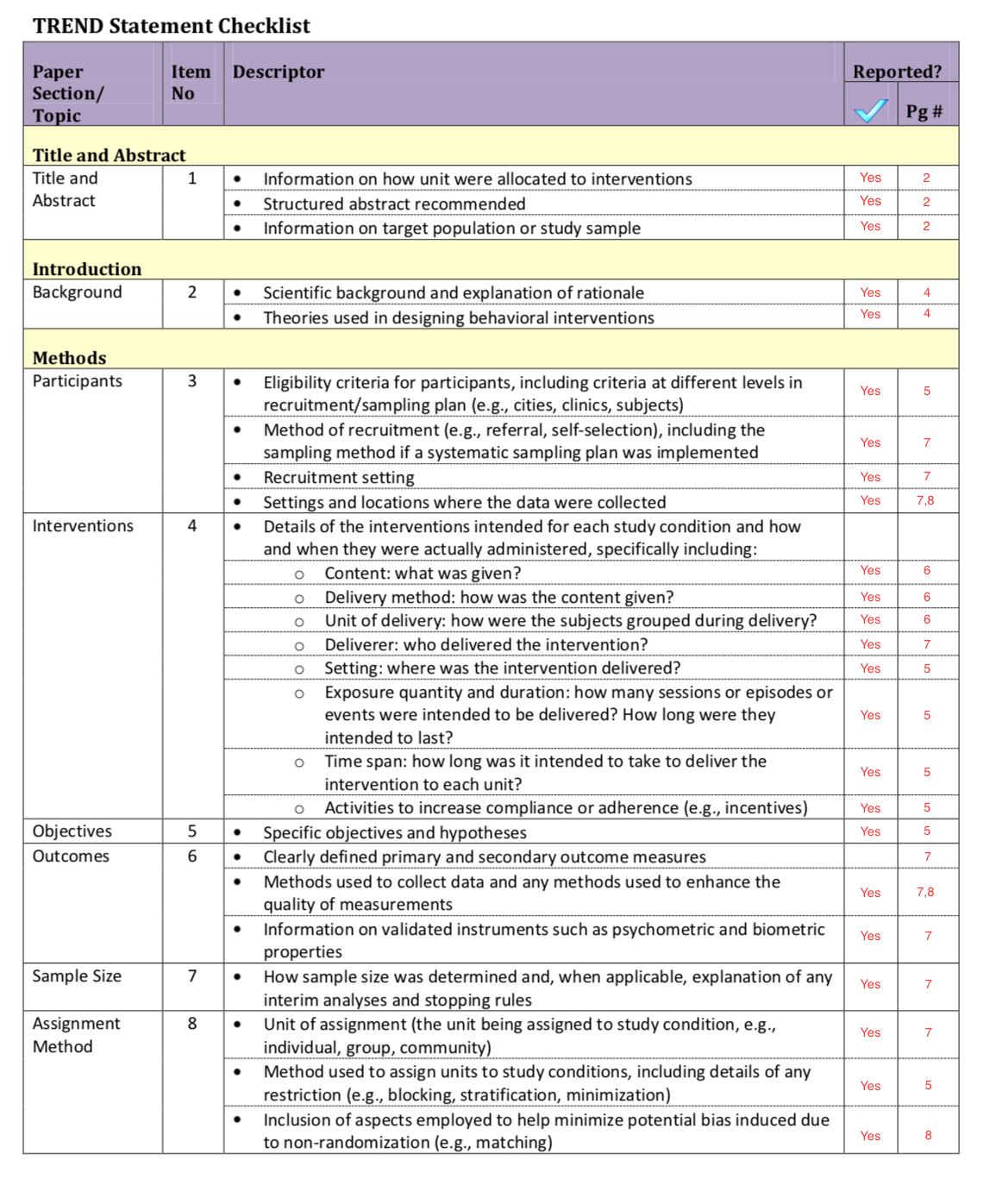


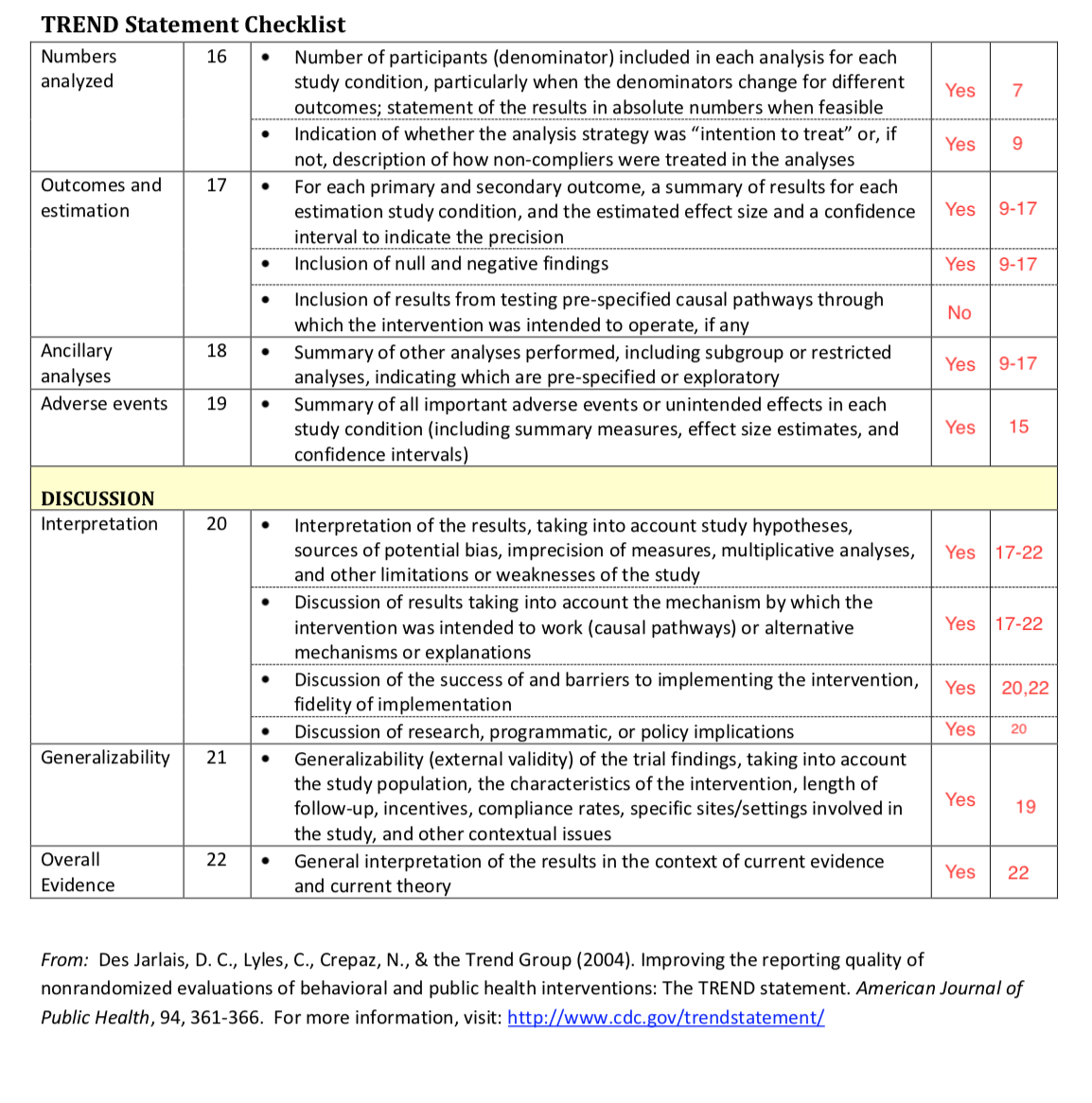

Supplement: S1 Checklist — (DOCX) [file pone.0273886.s001.docx]
