# Supplementary material for: Evaluation of the efficacy of using indocyanine green associated with fluorescence in sentinel lymph node biopsy
Source: PLoS One. 2023 Oct 25;18(10):e0273886. doi: 10.1371/journal.pone.0273886 (PMC10599532; doi:10.1371/journal.pone.0273886)
Supplement: S1 File — (DOCX) [file pone.0273886.s002.docx]

| **Número da paciente**  **incluída na pesquisa** | **Azul patente (n=33)** | **Azul patente + verde indocianina (n=33)** | **Verde indocianina (n=33)** |
| --- | --- | --- | --- |
| **1** | R$3638,35 |  |  |
| 2 | R$ 3888,43 |  |  |
| 3 | R$3888,43 |  |  |
| 4 | R$ 5930,26 |  |  |
| **5** | R$ 3654,44 |  |  |
| 6 | R$ 3912,43 |  |  |
| 7 | R$ 3992,23 |  |  |
| 8 | R$ 4573,25 |  |  |
| **9** | R$ 3993,43 |  |  |
| 10 | R$ 3841,00 |  |  |
| 11 | R$ 3832,23 |  |  |
| 12 | R$ 3920,23 |  |  |
| 13 | R$ 3756,34 |  |  |
| 14 | R$ 3743,76 |  |  |
| 15 | R$ 3896,43 |  |  |
| 16 | R$ 3872,23 |  |  |
| 17 | R$ 3928,23 |  |  |
| 18 | R$ 4477,25 |  |  |
| 19 | R$ 3872,23 |  |  |
| 20 | R$ 3835,76 |  |  |
| 21 | R$ 4429,25 |  |  |
| 22 | R$ 3888,43 |  |  |
| 23 | R$ 3928,43 |  |  |
| 24 | R$ 3992,23 |  |  |
| 25 | R$ 3881,29 |  |  |
| 26 | R$ 3223,43 |  |  |
| 27 | R$ 5916,59 |  |  |
| 28 | R$ 1930,83 |  |  |
| 29 | R$ 3261,35 |  |  |
| 30 | R$ 4441,07 |  |  |
| 31 | R$ 3872,23 |  |  |
| 32 | R$ 7170,70 |  |  |
| 33 | R$ 3872,43 |  |  |
| 34 |  |  | R$ 2060,15 |
| 35 |  |  | R$ 3848,26 |
| 36 |  |  | R$ 3704,23 |
| 37 |  |  | R$ 3874,74 |
| 38 |  |  | R$ 2773,42 |
| 39 |  |  | R$ 3255,43 |
| 40 |  |  | R$ 1937,83 |
| 41 |  |  | R$ 3985,75 |
| 42 |  |  | R$ 3894,55 |
| 43 |  |  | R$ 3122,65 |
| 44 |  |  | R$ 3550,01 |
| 45 |  |  | R$ 3765,78 |
| 46 |  |  | R$ 3453,45 |
| 47 |  |  | R$ 3568,78 |
| 48 |  |  | R$ 2185,31 |
| 49 |  |  | R$ 3765,65 |
| 50 |  |  | R$ 3877,56 |
| 51 |  |  | R$ 3887,56 |
| 52 |  |  | R$ 3978,56 |
| 53 |  |  | R$ 4459,17 |
| 54 |  |  | R$ 3976,78 |
| 55 |  |  | R$ 3765,45 |
| 56 |  |  | R$ 3224,22 |
| 57 |  |  | R$ 3678,76 |
| 58 |  |  | R$ 3955,76 |
| 59 |  |  | R$ 3267,76 |
| 60 |  |  | R$ 3568,45 |
| 61 |  |  | R$ 3987,43 |
| 62 |  |  | R$ 3975,94 |
| 63 |  |  | R$ 3765,87 |
| 64 |  |  | R$ 2563,44 |
| 65 |  |  | R$ 3873,89 |
| 66 |  |  | R$ 4006,96 |
| 67 |  | R$ 3874,45 |  |
| 68 |  | R$ 3754,65 |  |
| 69 |  | R$ 4107,53 |  |
| 70 |  | R$ 4039,45 |  |
| 71 |  | R$ 3423,43 |  |
| 72 |  | R$ 3777,43 |  |
| 73 |  | R$ 3489,56 |  |
| 74 |  | R$ 3872,23 |  |
| 75 |  | R$ 3522,56 |  |
| 76 |  | R$ 2916,94 |  |
| 77 |  | R$ 6371,44 |  |
| 78 |  | R$ 3755,37 |  |
| 79 |  | R$ 3544,76 |  |
| 80 |  | R$ 4823,15 |  |
| 81 |  | R$ 4675,24 |  |
| 82 |  | R$ 3276,44 |  |
| 83 |  | R$ 4716,92 |  |
| 84 |  | R$ 4716,02 |  |
| 85 |  | R$ 4463,34 |  |
| 86 |  | R$ 3908,21 |  |
| 87 |  | R$ 3942,33 |  |
| 88 |  | R$ 4626,46 |  |
| 89 |  | R$ 4504,91 |  |
| 90 |  | R$ 3553,56 |  |
| 91 |  | R$ 3723,45 |  |
| 92 |  | R$ 4607,60 |  |
| 93 |  | R$ 3512,23 |  |
| 94 |  | R$ 4491,79 |  |
| 95 |  | R$ 3754,76 |  |
| 96 |  | R$ 3892,42 |  |
| 97 |  | R$ 6325,56 |  |
| 98 |  | R$ 4835,95 |  |
| 99 |  | R$ 4047,45 |  |
