# Supplementary material for: Evaluation of the efficacy of using indocyanine green associated with fluorescence in sentinel lymph node biopsy
Source: PLoS One. 2023 Oct 25;18(10):e0273886. doi: 10.1371/journal.pone.0273886 (PMC10599532; doi:10.1371/journal.pone.0273886)
Supplement: S2 File — (DOCX) [file pone.0273886.s003.docx]

**Evaluation of the efficacy of using Indocyanine Green associated with fluorescence in sentinel lymph node biopsy**

Project to obtain the title of Doctor of Science

Student: Rafael Sa M.Sc

Advisor: Afonso Celso Pinto Nazário, Ph.D

**SUMMARY**

INTRODUCTION……………………………………………………..…….. 3

OBJECTIVES…………………………………………..…………..……… 4

MATERIALS AND METHODS…………….………………………..…… 5

EXPECTED OUTCOME……………………………………….…….…… 7

COSTS / EXPECTED DIFFICULTIES…………………………………. 8

TIMELINE……..…………………………………………………….…….. 9

REFERENCES…………………………………………………………… 10

1. **INTRODUCTION**

Sentinel lymph node biopsy is an established technique in the axillary staging of patients with breast cancer. Routine axillary lymphadenectomy has high morbidity, highlighting the following complications: seroma, local infection, paresthesia and lymphedema [1,2].

Randomized studies and meta-analyses have shown that negative sentinel biopsy has no impact on survival [3,4].

Three techniques are widely used globally for the detection of sentinel lymph nodes: patent blue, technetium 99 radiopharmaceutical with the use of the gamma probe and the combination of these two techniques [5-13].

Kitai et al was the first to report the technique known as ICG (Indocyaninae Green) in breast cancer and obtained an identification rate of 94%. ICG is a low molecular weight non-radioactive fluorescent dye. This chemical marker can penetrate human tissue to depths of a few millimeters to a few centimeters. This allows for real-time lymphatic migration, helping the surgeon to plan the dermal incision, thereby reducing the difficulty of the procedure[14-20].

**2) OBJECTIVES:**

**Primary:**

- To evaluate the sentinel lymph node detection rate with indocyanine green in breast cancer patients.

**Secondary:**

- To compare the sentinel lymph node detection rate in breast cancer patients using patent blue x indocyanine green x combined technique (patent blue + indocyanine green).
- To assess the economic impact of different sentinel lymph node detection techniques.

**3) MATERIALS AND METHODS**

**3.1) CASUISTRY**

Patients with breast cancer at the Hospital Regional do Câncer de Presidente Prudente (HRC) will be randomly subjected to one of three techniques to be studied in each arm of the study: patent blue, indocyanine green or combined (indocyanine + patent blue) .

These patients will be referred from the Basic Health Units to the Oncomastology Outpatient Clinic of the HRC via the Hebe Camargo Network (Regional Health Directorate) for the surgical treatment of breast cancer.

The inclusion criteria will be:

- All patients with breast cancer with surgical indication for sentinel lymph node biopsy with clinically negative armpits.

The exclusion criteria will be:

- Presence of T4, N1, N2 and N3 staging.
- Neoadjuvant chemotherapy.
- Allergy to iodine and its derivatives.

**3.2) METHODS**

Patients in the group using the well-known patent blue technique will undergo periareolar infiltration of 2 ml of this product with subsequent 5-minute breast massage for migration of the lymphotropic dye. After this period, during the axillary dissection, the lymphatic ducts will be visualized until the sentinel lymph node (first lymph node of the lymphatic drainage of the ipsilateral upper limb), which will be resected [1-3].

Patients in the innovative indocyanine green technique group will undergo periareolar infiltration of 5mg of this product, followed by breast massage for migration of the fluorescent dye. After this period, during axillary dissection, the lymphatic ducts up to the sentinel lymph node will be visualized using a fluorescence device from the German company Karlz-Storz® coupled to the VITOM II ICG (real-time visualization camera) which has an infrared light with 760 nm. The dermal incision and axillary dissection will be closer to the targeted lymph node, thus being more accurate [14-19].

Patients in the combined technique group will undergo the 2 procedures previously described [20].

**4) EXPECTED OUTCOME**

To prove the effectiveness of the use of the indocyanine green technique associated with the fluorescence device for the detection of sentinel lymph nodes, becoming an alternative to the use of the already established patent blue and technetium 99.

**5) EXPECTED COSTS AND DIFFICULTIES**

The patients studied will be from the Regional Cancer Hospital of Presidente Prudente, which already has the fluorescence device and will purchase the dye (indocyanine) for use in its patients. We hope to find few difficulties, since the Hospital Regional do Câncer de Presidente Prudente is a reference for breast oncology in the region of Oeste Paulista and only 3 surgeons from the same team will use the device.

**6) TIMELINE**

Project start: August/2019

End of project: December/2021

Delivery of study results: July/2022

**7) REFERÊNCIAS:**

[1] Veronesi U, Paganelli G, Viale G, Luini A, Zurrida S, Galimberti V, Intra M, Veronesi P, Robertson C, Maisonneuve P, Renne G, De Cicco C, De Lucia F, Gennari R: A randomized comparison of sentinel node biopsy with routine axillary dissection in breast cancer. N Eng J Med 2003, 349:546-553.

[2] Noguchi M: Sentinel lymph node biopsy as an alternative to routine axillary lymph node dissection in breast cancer patients. J Surg Oncol 2001, 76:144-156.

[3] Kim T, Giuliano AE, Lyman GH. Lymphatic mapping and sentinel lymph node biopsy in early-stage breast carcinoma: a metaanalysis. Cancer. 2006;106:4–16.

[4] Krag DN, Anderson SJ, Julian TB, Brown AM, Harlow SP, Cos- tantino JP, et al. Sentinel-lymph-node resection compared with conventional axillary-lymph-node dissection in clinically node- negative patients with breast cancer: overall survival findings from the NSABP B-32 randomised phase 3 trial. Lancet Oncol. 2010;11:927–33.

[5] Wada N, Imoto S, Yamauchi C, Hasebe T, Ochiai A, Ebihara S: Correlation between concordance of tracers, order of harvest, and presence of metastases in sentinel lymph nodes with breast cancer. Ann Surg Oncol 2005, 12:1-7. Kern KA: Concordance and validation study of sentinel lymph node biopsy for breast cancer using subareolar injection of blue dye and technetium 99 m sulfur colloid. J Am Coll Surg 2002, 195:467-475.

[6] Imoto S, Wada N, Murakami K, Hasebe T, Ochiai A, Ebihara S: Prognosis of breast cancer patients treated with sentinel node biopsy in Japan. Jpn J Clin Oncol 2004, 34:452-456.

[7] Tuttle TM: Technical advances in sentinel lymph node biopsy for breast cancer. Am Surg 2004, 70:407-413.

[8] Motomura K, Inaji H, Komoike Y, Hasegawa Y, Kasugai T, Noguchi S, Koyama H: Combination technique is superior to dye alone in identification of the sentinel node in breast cancer patients. J Surg Oncol 2001, 76:95-99.

[9] Tefra L, Lannin DR, Swanson MS, Van Eyk JJ, Verbanac KM, Chua AN, Ng PC, Edwards MS, Halliday BE, Henry CA, Sommers LM, Carman CM, Molin MR, Yurko JE, Perry RR, Williams R: Multicenter trial of sentinel node biopsy for breast cancer using both technetium sulfur colloid and isosulfan blue dye. Ann Surg 2001, 233:51-59.

[10] Derossis AM, Fey J, Yeung H, Yeh SDJ, Heerdt AS, Petrek J, VanZee KJ, Montgomery LL, Borgen PI, Cody HS III: A trend analysis of the relative value of blue dye and isotope localization in 2,000 consecutive cases of sentinel node biopsy for breast cancer. J Am Coll Surg 2001, 193:473-478.

[11] Krag DN, Weaver OJ, Alex JC, Fairbank JT: Surgical resection and radiolocalization of the sentinel lymph node in breast cancer using a gamma probe. Surg Oncol 1993, 2:335-340.

[12] Krag D, Weaver D, Ashikaga T, Moffat F, Klimberg S, Shriver C, Feldman S, Kusminsky R, Gadd M, Kuhn J, Harlow S, Beitsch P, Whitworth P, Foster R, Dowlatshahi K: The sentinel node in breast cancer-A multicenter validation study. N Engl J Med 1998, 339:941-946.

[13] Giuliano AE, Jones RC, Brennan M, Statman R: Sentinel lymphadenectomy in breast cancer. J Clin Oncol 1997, 15:2345-2350.

[14] Kitai T, Inomoto T, Miwa M, Shikayama T. Fluorescence navigation with indocyanine green for detecting sentinel lymph nodes in breast cancer. *Breast Cancer*. 2005;12:211–215.

[15] Hünerbein M, Kneif S, Mohr Z, Murawa D, Hirche C. ICG fluorescence-guided sentinel node biopsy for axillary nodal staging in breast cancer. Breast Cancer Res Treat 2010, 121:373-378.

[16] Tani T, Kurumi Y, Kubota Y, Cho H, Shimizu T, Kawai Y, Tanaka M, Umeda T, Mori T, Abe H. Indocyanine green fluorescence imagin system for senti lymph node biopsies in early breast cancer patients. Sure Today 2011, 41:197-202.

[17] Hirche, C, Kneser U, Hünerbein M, Engel H, Yang W, Gazyakan E, Xiong L. Indocyanine green fluorescence-guided sentinel node biopsy: A meta-analysis on detection rate and diagnostic performance. EJSO The Journal of Cancer Surgery 2014 1-7.

[18] Grischke EM, Ro ̈hm C, Hahn M et al (2015) ICG fluorescence technique for the detection of sentinel lymph nodes in breast cancer: results of a prospective open-label clinical trial. Geburt-shilfe Frauenheilkd 75:935–940.

[19] Boni L, David G, Mangano A et al (2015) Clinical applications of indocyanine green (ICG) enhanced fluorescence in laparoscopic surgery. Surg Endosc 29:2046–2055.

[20] Kinoshita T, Akashi S, Kikuyama M, Nagao T, Hojo T. Evaluation of sentinel node biopsy by combined fluorescent and dye method and lymph flow for breast cancer. The Breast 2010, 210-213.
