# Supplementary material for: Evaluation of the efficacy of using indocyanine green associated with fluorescence in sentinel lymph node biopsy
Source: PLoS One. 2023 Oct 25;18(10):e0273886. doi: 10.1371/journal.pone.0273886 (PMC10599532; doi:10.1371/journal.pone.0273886)
Supplement: S3 File — (DOCX) [file pone.0273886.s004.docx]

**Avaliação da eficácia do uso do Verde Indocianina associada à fluorescência na biópsia do linfonodo sentinela**

Projeto para obtenção do titulo de Doutor em Ciências

Aluno: Rafael Sá

Orientador: Prof. Dr. Afonso Celso Pinto Nazário

**SUMÁRIO**

INTRODUÇÃO……………………………………………………..…….. 3

OBJETIVOS………………………………………………………..……… 4

MATERIAIS E MÉTODOS…………….……………………..……..…… 5

RESULTADO ESPERADO……………………………………….……… 7

CUSTOS / DIFICULDADES ESPERADAS……………………………. 8

CRONOGRAMA………………………………………………………….. 9

REFERÊNCIAS…………………………………………………………… 10

1. **INTRODUÇÃO**

A biópsia do linfonodo sentinela é uma técnica consagrada no estadiamento axilar das pacientes portadoras de câncer de mama. A linfonodectomia axilar rotineira apresenta alta morbidade, destacando-se as seguintes complicações: seroma, infecção local, parestesia e linfedema [1,2].

Estudos randomizados e metanálises têm mostrado que a biópsia do sentinela negativa não tem impacto na sobrevida [3,4].

Três técnicas são muito utilizadas globalmente para deteção do linfonodo sentinela: azul patente, radiofármaco tecnécio 99 com uso do gamma probe e a combinação dessas duas técnicas [5-13].

Kitai et al foi o primeiro a reportar a técnica conhecida por ICG (Indocyaninae Green) no câncer de mama e obteve a taxa de identificação de 94%. ICG é um corante fluorescente não radioativo com baixo peso molecular. Este marcador químico pode penetrar no tecido humano em profundidades de poucos milímetros até alguns centímetros. Isto permite a migração linfática em tempo real, ajudando o cirurgião a planejar a incisão dérmica, deste modo reduzindo a dificuldade do procedimento [14-20].

**2) OBJETIVOS:**

**Primário:**

- Avaliar a taxa de detecção do linfonodo sentinela com verde de indocianina em pacientes com câncer de mama.

**Secundários:**

- Comparar a taxa de detecção do linfonodo sentinela em pacientes com câncer de mama usando as técnicas com azul patente x verde de indocianina x técnica combinada (azul patente + verde de indocianina).
- Avaliar o impacto econômico das diferentes técnicas de detecção do linfonodo sentinela.

**3) MATERIAIS E MÉTODOS**

**3.1) CASUÍSTICA**

As pacientes portadoras de câncer de mama do Hospital Regional do Câncer de Presidente Prudente (HRC) serão submetidas de forma aleatória a uma das três técnicas a serem estudadas em cada braço do estudo: azul patente, verde indocianina ou combinada (indocianina + azul patente).

Estas pacientes serão encaminhadas das Unidades Básicas de Saúde ao Ambulatório de Oncomastologia do HRC via Rede Hebe Camargo (Diretoria Regional de Saúde) para o tratamento cirúrgico do câncer de mama.

Os critérios de inclusão serão:

- Todas pacientes portadoras de câncer de mama com indicação cirúrgica de biópsia do linfonodo sentinela com axilas clinicamente negativas.

Os critérios de exclusão serão:

- Presença de estadiamento T4, N1, N2 e N3.
- Quimioterapia neo-adjuvante
- Alergia ao iodo e seus derivados.

**3.2) MÉTODOS**

As pacientes do grupo da conhecida técnica do azul patente serão submetidas à infiltração periareolar de 2 ml deste produto com posterior massagem mamária de 5 minutos para migração do corante linfotrópico. Após este período, durante a dissecção axilar, serão visualizados os ductos linfáticos até o linfonodo sentinela (primeiro linfonodo da drenagem linfática do membro superior ipsilateral), que será ressecado [1-3].

As pacientes do grupo da inovadora técnica do verde indocianina serão submetidas à infiltração periareolar de 5mg deste produto, seguida de massagem mamária para migração do corante fluorescente. Após este período, durante a dissecção axilar, serão visualizados os ductos linfáticos até o linfonodo sentinela através do uso do aparelho de fluorescência da empresa alemã Karlz-Storz® acoplado ao VITOM II ICG (câmera de visualização em tempo real) que possui luz infravermelha com 760 nm. A incisão dérmica e dissecção axilar serão mais próximas ao linfonodo almejado, sendo assim mais precisa [14-19].

As pacientes do grupo com a técnica combinada serão submetidas aos 2 procedimentos descritos previamente [20].

**4) RESULTADO ESPERADO**

Comprovar a eficácia do uso da técnica do verde indocianina associado ao aparelho de fluorescência para a detecção do linfonodo sentinela, tornando-se uma alternativa em relação ao uso dos já consagrados azul patente e tecnécio 99.

**5) CUSTOS E DIFICULDADES ESPERADAS**

As pacientes estudadas serão do Hospital Regional do Câncer de Presidente Prudente, que já possui o aparelho de fluorescência e irá realizar a compra do corante (indocianina) para uso em suas pacientes.

Esperamos encontrar poucas dificuldades, uma vez que o Hospital Regional do Câncer de Presidente Prudente é referência para oncologia mamária na região do Oeste Paulista e apenas 3 cirurgiões da mesma equipe irão utilizar o aparelho.

**6) CRONOGRAMA**

Início do projeto : Agosto/2019

Fim do projeto : Dezembro/2021

Entrega dos resultados do estudo : Julho/2022

**7) REFERÊNCIAS:**

[1] Veronesi U, Paganelli G, Viale G, Luini A, Zurrida S, Galimberti V, Intra M, Veronesi P, Robertson C, Maisonneuve P, Renne G, De Cicco C, De Lucia F, Gennari R: A randomized comparison of sentinel node biopsy with routine axillary dissection in breast cancer. N Eng J Med 2003, 349:546-553.

[2] Noguchi M: Sentinel lymph node biopsy as an alternative to routine axillary lymph node dissection in breast cancer patients. J Surg Oncol 2001, 76:144-156.

[3] Kim T, Giuliano AE, Lyman GH. Lymphatic mapping and sentinel lymph node biopsy in early-stage breast carcinoma: a metaanalysis. Cancer. 2006;106:4–16.

[4] Krag DN, Anderson SJ, Julian TB, Brown AM, Harlow SP, Cos- tantino JP, et al. Sentinel-lymph-node resection compared with conventional axillary-lymph-node dissection in clinically node- negative patients with breast cancer: overall survival findings from the NSABP B-32 randomised phase 3 trial. Lancet Oncol. 2010;11:927–33.

[5] Wada N, Imoto S, Yamauchi C, Hasebe T, Ochiai A, Ebihara S: Correlation between concordance of tracers, order of harvest, and presence of metastases in sentinel lymph nodes with breast cancer. Ann Surg Oncol 2005, 12:1-7. Kern KA: Concordance and validation study of sentinel lymph node biopsy for breast cancer using subareolar injection of blue dye and technetium 99 m sulfur colloid. J Am Coll Surg 2002, 195:467-475.

[6] Imoto S, Wada N, Murakami K, Hasebe T, Ochiai A, Ebihara S: Prognosis of breast cancer patients treated with sentinel node biopsy in Japan. Jpn J Clin Oncol 2004, 34:452-456.

[7] Tuttle TM: Technical advances in sentinel lymph node biopsy for breast cancer. Am Surg 2004, 70:407-413.

[8] Motomura K, Inaji H, Komoike Y, Hasegawa Y, Kasugai T, Noguchi S, Koyama H: Combination technique is superior to dye alone in identification of the sentinel node in breast cancer patients. J Surg Oncol 2001, 76:95-99.

[9] Tefra L, Lannin DR, Swanson MS, Van Eyk JJ, Verbanac KM, Chua AN, Ng PC, Edwards MS, Halliday BE, Henry CA, Sommers LM, Carman CM, Molin MR, Yurko JE, Perry RR, Williams R: Multicenter trial of sentinel node biopsy for breast cancer using both technetium sulfur colloid and isosulfan blue dye. Ann Surg 2001, 233:51-59.

[10] Derossis AM, Fey J, Yeung H, Yeh SDJ, Heerdt AS, Petrek J, VanZee KJ, Montgomery LL, Borgen PI, Cody HS III: A trend analysis of the relative value of blue dye and isotope localization in 2,000 consecutive cases of sentinel node biopsy for breast cancer. J Am Coll Surg 2001, 193:473-478.

[11] Krag DN, Weaver OJ, Alex JC, Fairbank JT: Surgical resection and radiolocalization of the sentinel lymph node in breast cancer using a gamma probe. Surg Oncol 1993, 2:335-340.

[12] Krag D, Weaver D, Ashikaga T, Moffat F, Klimberg S, Shriver C, Feldman S, Kusminsky R, Gadd M, Kuhn J, Harlow S, Beitsch P, Whitworth P, Foster R, Dowlatshahi K: The sentinel node in breast cancer-A multicenter validation study. N Engl J Med 1998, 339:941-946.

[13] Giuliano AE, Jones RC, Brennan M, Statman R: Sentinel lymphadenectomy in breast cancer. J Clin Oncol 1997, 15:2345-2350.

[14] Kitai T, Inomoto T, Miwa M, Shikayama T. Fluorescence navigation with indocyanine green for detecting sentinel lymph nodes in breast cancer. *Breast Cancer*. 2005;12:211–215.

[15] Hünerbein M, Kneif S, Mohr Z, Murawa D, Hirche C. ICG fluorescence-guided sentinel node biopsy for axillary nodal staging in breast cancer. Breast Cancer Res Treat 2010, 121:373-378.

[16] Tani T, Kurumi Y, Kubota Y, Cho H, Shimizu T, Kawai Y, Tanaka M, Umeda T, Mori T, Abe H. Indocyanine green fluorescence imagin system for senti lymph node biopsies in early breast cancer patients. Sure Today 2011, 41:197-202.

[17] Hirche, C, Kneser U, Hünerbein M, Engel H, Yang W, Gazyakan E, Xiong L. Indocyanine green fluorescence-guided sentinel node biopsy: A meta-analysis on detection rate and diagnostic performance. EJSO The Journal of Cancer Surgery 2014 1-7.

[18] Grischke EM, Ro ̈hm C, Hahn M et al (2015) ICG fluorescence technique for the detection of sentinel lymph nodes in breast cancer: results of a prospective open-label clinical trial. Geburt-shilfe Frauenheilkd 75:935–940.

[19] Boni L, David G, Mangano A et al (2015) Clinical applications of indocyanine green (ICG) enhanced fluorescence in laparoscopic surgery. Surg Endosc 29:2046–2055.

[20] Kinoshita T, Akashi S, Kikuyama M, Nagao T, Hojo T. Evaluation of sentinel node biopsy by combined fluorescent and dye method and lymph flow for breast cancer. The Breast 2010, 210-213.
