# Supplementary material for: Evaluation of the efficacy of using indocyanine green associated with fluorescence in sentinel lymph node biopsy
Source: PLoS One. 2023 Oct 25;18(10):e0273886. doi: 10.1371/journal.pone.0273886 (PMC10599532; doi:10.1371/journal.pone.0273886)
Supplement: Attachment — Submitted filename: Response to Reviewers.docx [file pone.0273886.s007.docx]

**Reviewer 1**

**Evaluations (peer review comments for the author)**

**Reviewer #1: A non-randomized, prospective clinical trial was conducted which aimed to evaluate the efficacy and the sentinel lymph node detection rate when using indocyanine green, an innovative technique. The accuracy rates were 78.8%, 93.9%, and 100% for patent blue, indocyanine green and the combination of the two, respectively.**

**Minor revisions:**

**1- Within the abstract and the main section, provide 95% confidence intervals for the detection rates and standard deviations for the mean times of sentinel lymph node detection and surgery.**

RESPONSE: Thank you for the observation. Considering that the IC is only valid when both np and nq are higher than ten or npq > 5, we decided not to present the 95% confidence intervals for the detection rates because it didn't occur in our data. Thus, we opted only to show the observed rates, which are still relevant. The standard deviation was presented alongside its mean time, as per request.

**2- Statistical analysis section: Clarify what is meant by “To compare the values spent . . .”.**

RESPONSE: We compared the total hospitalization costs of each patient participating in the research, including the dye, to assess the possible socioeconomic impact of using a certain dye.

**3- Before applying Tukey’s multiple comparison tests, check for a significant overall effect. For example, compare the three means using an ANOVA test (overall test), if it is significant then apply Tukey’s multiple comparison tests. If it is not significant, do not apply Tukey’s tests.**

RESPONSE: The multiple comparison tests occurred as described by the reviewer. The p-values presented in the table refer to the ANOVA, and the subscripted letters identify the groups with significant differences according to Tukey's test. Thus, we first applied the ANOVA, and only when the result was significant, we used Tukey's multiple comparison test. To avoid visually overloading the table, we used the letters to identify the groups with significant statistical differences.

**4- The standard statistical term for average is mean.**

RESPONSE: This was adjusted in the text and table, thank you.

**5- Data is missing in table 3 for the combination of dyes.**

RESPONSE: We thank the reviewer for noticing this. This missing data has already been correctly filled in in Table 3.

**6- Tukey's test is used for pairwise comparisons. Thus this statement is unclear because the pairs have not been identified. “*According to the p-values of Tukey's multiple comparison test, p=0.013 for the blue-green comparison, p=0.003 for the combined green, and 0.905 for the combined blue.”**

RESPONSE: We adjusted table 3 to keep the same p-value presentation pattern of table 2. The p-value refers to the ANOVA test, and the letters identify statistically different groups, as per Tukey's test. There were no significant differences between the combined and blue dyes, so we omitted the letter A.

**7- Figure 2: Add error bars for each group at the two time points.**

RESPONSE: We did not add error bars to the chart for the same reason cited in the item's one response, considering that error bars usually represent standard error (SE).

**8- Table 2: Identify the statistical testing method used to estimate each p-value. Since all continuous factors are summarized as means and standard deviations, are all data normally distributed and all p-values from an ANOVA test? If these values are non-normally distributed, summarize using median, first and third quartiles and compare with the Kruskal-Wallis test.**

RESPONSE: We adjusted table 2 to clarify the method of p-value estimation. The only variable for which we used the Kruskall-Wallis test was the number of identified lymph nodes (to compare the mean group). When the dye comparison was presented using Qui-Square, we removed the line comparing the means. Thus, the ANOVA test compared all quantitative variables between the groups, while multiple comparisons were through Tukey's test. Before applying the ANOVA test, we tested the normality of the data through the Shapiro-Wilk test.

**9- To assist in the review process, add line numbering to the document.**

RESPONSE: We added line numbering in the manuscript to assist in the review process; thank you for this suggestion.

**Reviewer 2**

**Evaluations (peer review comments for the author)**

**Reviewer #2: The manuscript investigated the sentinel lymph node detection rate between the use of patent blue, indocyanine green and patent blue + indocyanine green (combined). Although some similar researches have already published, the specific local studies in Brazil further prove the ICG for the use of sentinel lymph nodes with results better than patent blue. The experimental design was sound, with reasonable data interpretation and result discussion. However, some specific points should be stated clearly as follow**：

1.**The patients and tumor characteristics should provide as tables in the manuscript.**

RESPONSE: Thank you for the observation. We added the information to the two new tables (Table 1 and Table 3).

**2. The abbreviation ICG of indocyanine green could used directly in the manuscript except for the first explanation.**

RESPONSE: Thank you for the suggestion. We updated the manuscript with the abbreviation.

**3. In section "Materials used", the authors should check the description of "indocyanine green dye 5ml/10ml". It should be “5 mg/10 mL”?**

RESPONSE: Exactly. We apologize for this error and have already corrected this unit in the text.

**4. Language has to be improved to make the article more attractive.**

RESPONSE: We had the language reviewed. We thank the reviewer for the collaboration with our manuscript.

**Editorial Corrections (Journal requirements):**

1. Please ensure that your manuscript meets PLOS ONE's style requirements, including those for file naming. The PLOS ONE style templates can be found at <https://journals.plos.org/plosone/s/file?id=wjVg/PLOSOne_formatting_sample_main_body.pdf> and <https://journals.plos.org/plosone/s/file?id=ba62/PLOSOne_formatting_sample_title_authors_affiliations.pdf>.

RESPONSE: We edited and improved the manuscript with the PLOS ONE's style requirements.

2. Thank you for stating the following in the Acknowledgments Section of your manuscript: “The first author (RSS) was supported as a Doctoral Scholar by the National Council for Scientific and Technological Development (CNPq). This study received no other funding.” We note that you have provided funding information that is not currently declared in your Funding Statement. However, funding information should not appear in the Acknowledgments section or other areas of your manuscript. We will only publish funding information present in the Funding Statement section of the online submission form. Please remove any funding-related text from the manuscript and let us know how you would like to update your Funding Statement. Currently, your Funding Statement reads as follows: “RSS 1 National Council for Scientific and Technological Development (CNPq) <https://www.gov.br/cnpq/pt-br>. The funders had no role in study design, data collection and analysis, decision to publish, or preparation of the manuscript.” Please include your amended statements within your cover letter; we will change the online submission form on your behalf.

RESPONSE: We corrected the acknowledgments section.

3. We note that you have indicated that data from this study are available upon request. PLOS only allows data to be available upon request if there are legal or ethical restrictions on sharing data publicly. For information on unacceptable data access restrictions, please see <http://journals.plos.org/plosone/s/data-availability#loc-unacceptable-data-access-restrictions>. In your revised cover letter, please address the following prompts: a) If there are ethical or legal restrictions on sharing a de-identified data set, please explain them in detail (e.g., data contain potentially identifying or sensitive patient information) and who has imposed them (e.g., an ethics committee). Please also provide contact information for a data access committee, ethics committee, or other institutional body to which data requests may be sent. b) If there are no restrictions, please upload the minimal anonymized data set necessary to replicate your study findings as either Supporting Information files or to a stable, public repository and provide us with the relevant URLs, DOIs, or accession numbers. Please see http://www.bmj.com/content/340/bmj.c181.long for guidelines on how to de-identify and prepare clinical data for publication. For a list of acceptable repositories, please see <http://journals.plos.org/plosone/s/data-availability#loc-recommended-repositories>. We will update your Data Availability statement on your behalf to reflect the information you provide.

RESPONSE: We are providing the anonymized data upload set necessary to replicate your study findings. This study does not have an ethical or legal restriction on sharing an unidentified dataset. You can find the data attached in a separate file.
