# Supplementary material for: Evaluation of the efficacy of using indocyanine green associated with fluorescence in sentinel lymph node biopsy
Source: PLoS One. 2023 Oct 25;18(10):e0273886. doi: 10.1371/journal.pone.0273886 (PMC10599532; doi:10.1371/journal.pone.0273886)
Supplement: Attachment — Submitted filename: Response to Reviewers.docx [file pone.0273886.s008.docx]

Hugh Cowley, Staff Editor
PLOS ONE

Dear Hugh Cowley

Thank you for your consideration of our paper for publication in PLOS ONE, and also for the positive reviews.

We specially thank Reviewer #3 for the careful review of the manuscript, and we would like them to know that we had the text professionally edited, with all the corrections listed made and also further improvements. We believe the paper is now ready for publication.

Please let us know if you have any other questions.

Thank you very much

Rafael S. Sá and co-authors

Reviewers' comments:

Reviewer's Responses to Questions

**Comments to the Author**

1. If the authors have adequately addressed your comments raised in a previous round of review and you feel that this manuscript is now acceptable for publication, you may indicate that here to bypass the “Comments to the Author” section, enter your conflict of interest statement in the “Confidential to Editor” section, and submit your "Accept" recommendation.

Reviewer #1: All comments have been addressed

Reviewer #3: (No Response)

**RESPONSE: Thank you for the positive evaluation. We have corrected all issues raised in the last review, and went further with a text editing and more corrections.**

2. Is the manuscript technically sound, and do the data support the conclusions?

The manuscript must describe a technically sound piece of scientific research with data that supports the conclusions. Experiments must have been conducted rigorously, with appropriate controls, replication, and sample sizes. The conclusions must be drawn appropriately based on the data presented.

Reviewer #1: (No Response)

Reviewer #3: Partly

**RESPONSE: As mentioned above, we have addressed all issues raised in the last review, and went further with a text editing and more corrections. This made the text clearer and we are sure that Reviewer #3 can now be confident about the robustness of our research methods and reporting.**

3. Has the statistical analysis been performed appropriately and rigorously?

Reviewer #1: (No Response)

Reviewer #3: Yes

4. Have the authors made all data underlying the findings in their manuscript fully available?

The [PLOS Data policy](http://www.plosone.org/static/policies.action#sharing) requires authors to make all data underlying the findings described in their manuscript fully available without restriction, with rare exception (please refer to the Data Availability Statement in the manuscript PDF file). The data should be provided as part of the manuscript or its supporting information, or deposited to a public repository. For example, in addition to summary statistics, the data points behind means, medians and variance measures should be available. If there are restrictions on publicly sharing data—e.g. participant privacy or use of data from a third party—those must be specified.

Reviewer #1: (No Response)

Reviewer #3: Yes

**RESPONSE: Thank you for the positive reviews in items 3 and 4 above.**

5. Is the manuscript presented in an intelligible fashion and written in standard English?

PLOS ONE does not copyedit accepted manuscripts, so the language in submitted articles must be clear, correct, and unambiguous. Any typographical or grammatical errors should be corrected at revision, so please note any specific errors here.

Reviewer #1: (No Response)

Reviewer #3: No

**RESPONSE: As mentioned above, we have hired professional text editing and made more corrections to the text. This made the text content clearer and the reading flows better now.**

6. Review Comments to the Author

Please use the space provided to explain your answers to the questions above. You may also include additional comments for the author, including concerns about dual publication, research ethics, or publication ethics. (Please upload your review as an attachment if it exceeds 20,000 characters)

Reviewer #1: (No Response)

Reviewer #3: This study compared 3 different methods to detect the axillary sentinel lymph nodes in breast cancer surgery; blue dye alone, ICG fluorescence alone, and their combination. They reported the significant improvement in the detection rate and procedure time of sentinel node biopsy when ICG fluorescence was used. Although the study was relatively small, with 33 cases in each group, and was a serial and retrospective comparison, the findings were very encouraging considering it was the first study to demonstrated the feasibility of ICG fluorescent method in Brazilian community. I sincerely respect the authors for their massive efforts and enthusiasm, and totally agreed that this kind of data could be important basis for the implementation of ICG fluorescence into Brazilian society. However, I found a substantial number of grammatical errors in the manuscript. In addition, the style of the manuscript was not organized well as a scientific manuscript. Thus, in a lot of parts of the manuscript, I could not follow what the authors really wanted to address. The following are examples which should to be corrected. Collectively, I found the manuscript was basically encouraging but not to be accepted in its current form. I strongly recommend the authors have it professionally edited and submit it as a separate manuscript.

- Page6 line 4: “patent blue dye v 50mg/2ml”. “v” does not make sense.

- Page6 line7: “5ml/10ml” might be correctly “5mg/10ml”
- Page6 line8; “periareolar infltration” should be “periareolar injection”
- Page6 line 10-11: I think this sentence contains multiple grammatical errors.
- Page6 line 19: The term “axillary dissection” is misleading since it normally means axillary cleanance.
- Page6 line22: “10ml/5mg” should be “5mg/10ml”.
- Page7 line6: Although I can follow what the author wants to say here, the sentence looks wired.
- Page8 line 3: the sentence doesn’t make sense.
- Page8 line7 to 16: this part doesn’t make sense.
- Page8 line21: “Time of identification” should be “Time to identification”.
- Page10 line6; ”menacme” might be “menarche”.
- From page 11: please separate the tables and figures from the body of manuscript.
- Page 11 to 12: the contents written in S2 Table should not be addressed repeatedly in the body of manuscripts.
- Page12 line 10: Please provide the definition of “accuracy rate”.
- Page14 line 7: “amounts spent” does not make sense.
- Page14 line 10: please spell out “BRL” when it firstly appears in the manuscript.
- Page15 1st section of the discussion: I agreed with the authors’ statement.

**RESPONSE: During the professional editing we hired for this manuscript, all items above have been corrected and the info added to the manuscript, except for thre issues:**

**1) the word menacme, which is correct in the context of our study. Menarche is defined as the first menstrual period in a woman’s life, which does not apply here. In our study, almost 30% of women were in the menacme phase, meaning “the period of a woman’s life cycle in which she experiences menstrual activity”. Reference for this definition here:** [**https://dictionary.apa.org/menacme**](https://dictionary.apa.org/menacme)**.**

**2) we do not understand what the reviewer means by “S2 Table” — we believe that all tables should be cited in the text, and this is what we tried to do.**

**3) We did not remove the tables from the manuscript document, as this is required in the journal’s Instructions for Authors: “Place each table in your manuscript file directly after the paragraph in which it is first cited (read order)”.**

7. PLOS authors have the option to publish the peer review history of their article ([what does this mean?](https://journals.plos.org/plosone/s/editorial-and-peer-review-process#loc-peer-review-history)). If published, this will include your full peer review and any attached files.
If you choose “no”, your identity will remain anonymous but your review may still be made public.
**Do you want your identity to be public for this peer review?** For information about this choice, including consent withdrawal, please see our [Privacy Policy](https://www.plos.org/privacy-policy).

Reviewer #1: No

Reviewer #3: No
