# Supplementary material for: Evaluation of the efficacy of using indocyanine green associated with fluorescence in sentinel lymph node biopsy
Source: PLoS One. 2023 Oct 25;18(10):e0273886. doi: 10.1371/journal.pone.0273886 (PMC10599532; doi:10.1371/journal.pone.0273886)
Supplement: Attachment — Submitted filename: Response to Reviewers.docx [file pone.0273886.s009.docx]

**PONE-D-22-22937R2**

**Evaluation of the efficacy of using indocyanine green associated with fluorescence in sentinel lymph node biopsy**

**PLOS ONE**

**Dear Salih Colakoglu - Academic Editor, PLOS ONE**

**Thank you for considering our manuscript for publication in PLOS-ONE. We appreciate the time and valuable suggestions from Reviewers #1 and #3.**

**We are responding to all reviewers’ queries below and we made changes to the manuscript accordingly, including text, tables and figures as requested. We believe the paper is now ready for publication, but if any point still needs modifications, please let us know.**

**Sincerely,**

**The authors**

Reviewers’ questions 1-5 and 7 don’t need authors’ responses.

6. Review Comments to the Author

Reviewer #1: Minor revisions:
1- Line 48: After "mean" add "+/- SD (standard deviation)." **– CORRECTED, THANK YOU.**
2- Line 52: Indicate the statistical testing method used to estimate p<0.001. **– INFORMATION ADDED, THANK YOU.**

3- Line 207: For clarity, simply state, "The data was collected in a Microsoft Excel spreadsheet prepared by the statistician." **– CORRECTED, THANK YOU.**
4- Line 308: Clarify: "normally distributed variables." **– CORRECTED, THANK YOU.**
5- Line 309: Clarify: "non-normally distributed variables." **– CORRECTED, THANK YOU.**
6- Line 315: Consider replacing the following statement: "The significance level adopted in all tests was 5%." with "P-values less than 0.05 were considered statistically significant." **– MODIFIED ACCORDING TO REVIEWER’S TASTE.**
7- The phrase "no report of male patients" in line 330 is awkward. **– CORRECTED, THANK YOU.**
8- Tables 1 and 3: Distinguish the row for age from those for menopausal status. **– CORRECTED, THANK YOU.**
9- Table 3: Merge the cells in the "BMI" header row to make it consistent with the rest of the table. **– CORRECTED, THANK YOU.**
10- Line 436: The standard statistical term for average is mean. **– MODIFIED ACCORDING TO REVIEWER’S TASTE.**
11- Figure 2: Label the y-axis: "Percentage".**– CORRECTED, THANK YOU.**
12- Figure 3: Label the y-axis. **– CORRECTED, THANK YOU.**
Note: Line numbers refer to those in the tracked changes version of revision 2.

Reviewer #3: Thanks for your re-submission. English has been improved and now I can review the manuscript in depth. I think the manuscript is still to be revised.

- First of all, I have some comments on Figures.
The unnecessary subtitle and the logo of Fig1 (CONSORT TRNAPARENT,,, and Evaluation of the efficacy,,,) should be removed. **– CORRECTED, THANK YOU.**
- In Fig 4, Circle chart is not appropriate to present this kind of data. Use bar chart or Table. I think the information of Fig2 and 4 should be presented by using Table. These may be able to incorporated into Table 4 or the new table with similar structure. **– THE DATA IS ALREADY PRESENTED IN TABLE 4, SO WE DELETED THE FIGURE.**

- Title of Fig 3 should be considered again, I suppose that many readers cannot understand what is the "rate" of sentinel lymph node identification time. And consider the style of the graph again. Dot plot with connecting bars does not fit for this kind of data. Use table if you cannot determine the proper style. **– THANK YOU FOR THE SUGGESTION. WE CREATED A BAR CHART AND ADDED IT TO THE MANUSCRIPT WITH A NEW TITLE.**

- Line 168: please correct the spacing of “5 mg/10 ml” **– SPACING HAS BEEN STANDARDIZED FOR ALL, THANK YOU.**
- Line 179, Line 185, and many others throughout the manuscript: As I mentioned before, please use "injection" rather than "infiltration". Most of the article use the term "injection". **– CORRECTED, THANK YOU.**
- Line 261: Similarly, the word "menacme" is not commonly used. Please using the term "premenopausal" is much better. **– MODIFIED ACCORDING TO REVIEWER’S PREFERENCE.**
- Line 182 and 189: As I mentioned before, since you did not perform ”axillary clearance” in many cases, you need to rephrase the term to avoid the reader’s confusion. For instance, I suggest the use the word “axillary surgery”. 0 **MODIFIED.**

- Line 355-367: There are a lot of trials evaluating the detection rate of ICG fluorescence in comparison with blue dye and its usefulness has been widely accepted although many clinical guidelines do not mention it. Therefore, the authors should cite at least the following meta-analysis and improve their discussion. In my understanding, this study is a kind of validation study of ICG fluorescence in Brazil. **– THANK YOU FOR THE SUGGESTION, WE ADDED TEXT CITING TWO OF THESE STUDIES.**
1. Lancet Oncol. 2014 Jul;15(8):e351-62. doi: 10.1016/S1470-2045(13)70590-4.
2. PLoS One. 2016 Jun 9;11(6):e0155597. doi: 10.1371/journal.pone.0155597. eCollection 2016.
3. Eur J Surg Oncol. 2014 Jul;40(7):843-9. doi: 10.1016/j.ejso.2014.02.228. Epub 2014 Feb 25.

- Line 365-367: Can the author compare the BMI or other factors between the patients whose sentinel node could be identified and those could not be identified? **– WE DID COMPARE AND FOUND NO SIGNIFICANT DIFFERENCE. THIS ANALYSIS WAS ADDED TO THE TEXT.**

- Line399-409: Why don’t you discuss about the concentration of ICG more precisely? You will be able to easily find that most of the trials use ICG at the concentration of 2.5mg/ml to 5mg/ml and injection dose is 0.5 ml to 1.0ml and reported good detection rate in SLNB. This issue has already discussed in a textbook; Yamagami, K, et al. Practice of Fluorescence Navigation Surgery Using Indocyanine Green for Sentinel Lymph Node Biopsy in Breast Cancer. In: Kusano, M., Kokudo, N., Toi, M., Kaibori, M. (eds) ICG Fluorescence Imaging and Navigation Surgery. Springer, Tokyo. 2016, pp 113–123. If the authors think this study is a kind of landmark trial in Brazil, such practical information should be provided here. **– WE THANK THE REVIEWER FOR THE SUGGESTION. WE READ THE STUDY AND DISCUSSED ITS FINDINGS IN THE MANUSCRIPT.**

- Line 451: In terms of long-term follow up, these two articles should be the largest ones rather than the report by Shen et al.
1. Breast Cancer Res Treat. 2021 Jul;188(2):361-368. doi: 10.1007/s10549-021-06196-6. Epub 2021 Mar 24
2. Int J Clin Oncol. 2021 Aug;26(8):1461-1468. doi: 10.1007/s10147-021-01925-9. Epub 2021 Apr 20 **– MODIFIED; STUDIES CITED, THANK YOU.**
- Line 300: Again, what is “accuracy rate”? Please give us the definition. It means detection rate? **– CORRECTED TO “DETECTION RATE”, THANK YOU.**

- Line 390-398: I think this part of the discussion is not relevant to this study. Is it necessary? **– WE DELETED THE PARAGRAPH.**

- Can authors provide the concordance rate between blue dye and ICG in the combination arm? In other word, how many nodes can be detected only by ICG or vice versa. – **THANK YOU FOR THE SUGGESTION, TABLE ADDED (AS TABLE 6).**

- Please discuss on the result of number of identified lymph node. It should be a key finding of this study but I could not find the discussion on the date in the manuscript. – **THANK YOU FOR THE SUGGESTION, TEXT ADDED TO THE DISCUSSION.**
- Conclusions: “The combined method was the most effective” is overstated. Your data shows ICG alone is also acceptable since it has a good performance in SLN identification and it can avoid tattooing. **– CORRECTED, THANK YOU.**
